# Taste Metaphors Ground Emotion Concepts Through the Shared Attribute of Valence

**DOI:** 10.3389/fpsyg.2022.938663

**Published:** 2022-07-12

**Authors:** Jason A. Avery, Alexander G. Liu, Madeline Carrington, Alex Martin

**Affiliations:** Laboratory of Brain and Cognition, National Institute of Mental Health, Bethesda, MD, United States

**Keywords:** taste, emotion, metaphor, cognition, grounded cognition

## Abstract

“Parting is such sweet sorrow.” Taste metaphors provide a rich vocabulary for describing emotional experience, potentially serving as an adaptive mechanism for conveying abstract emotional concepts using concrete verbal references to our shared experience. We theorized that the popularity of these expressions results from the close association with hedonic valence shared by these two domains of experience. To explore the possibility that this affective quality underlies the semantic similarity of these domains, we used a behavioral “odd-one-out” task in an online sample of 1059 participants in order to examine the semantic similarity of concepts related to emotion, taste, and color, another rich source of sensory metaphors. We found that the semantic similarity of emotion and taste concepts was greater than that of emotion and color concepts. Importantly, the similarity of taste and emotion concepts was strongly related to their similarity in hedonic valence, a relationship which was also significantly greater than that present between color and emotion. These results suggest that the common core of valence between taste and emotion concepts allows us to bridge the conceptual divide between our shared sensory environment and our internal emotional experience.

## Introduction

Metaphors such as “parting is such sweet sorrow”, “right to the bitter end” exemplify the use of our experience of taste to express our emotional states, which is naturally a difficult task, given the internal and essentially subjective nature of emotions. One reason for this phenomenon might have to do with the affective attributes of hedonic valence and arousal (physiological activation), shared by both domains of experience (Young, 1966; Russell, 2003; Small et al., 2003; Barrett and Bliss-Moreau, 2009; Sakamoto and Watanabe, 2016). Current theories of emotion posit that, like taste and interoception, the perception of the internal state of the body, emotion fundamentally serves as a homeostatic signaling mechanism, with certain highly valenced emotions such as fear promoting bodily homeostasis and social emotions such as loss and heartache potentially signaling deviations from homeostasis within the context of social relationships (Damasio, 1999; Barrett and Simmons, 2015). With some exceptions, sweet tastes have positive hedonic valence and bitter tastes negative, which are fundamental properties of food that individuals learn very early in development. Thus, when trying to communicate about other experiences with positive or negative valence, such as internal emotional experiences, we might naturally use terms relating to taste as external referents to convey the substance of our message.

An underlying reason for this shared association with valence might have to do with the neural structures shared by both taste and emotion, such as the insular cortex. The insular cortex is the primary cortical area involved in taste perception (Small, 2010; Avery et al., 2020) and in representing inferences about specific taste properties, which are automatically activated when viewing pictures of food (Simmons et al., 2013b; Avery et al., 2021). The insular cortex is also heavily implicated in the sensory component of emotional experience (Singer et al., 2009), perhaps due to its involvement in the representation of pain and interoceptive sensations from the body (Craig, 2003; Simmons et al., 2013a; Avery et al., 2017). Indeed, according to the Conceptual Metaphor Theory taste–emotion metaphors serve to ground the abstract emotional concepts by using a concrete reference to our shared experience (Lakoff and Johnson, 1980, 1999; Barsalou, 2008; Martin, 2016).

With such a close functional connection of taste and emotion in the human brain and given the shared importance of valence, we might expect that these cognitive domains are tightly linked within the conceptual structure of human thought. One way we can explore this possibility is through examining the semantic similarity of taste and emotion concepts present in the English language. We predict that taste and emotion concepts would exhibit a high degree of semantic similarity, as measured by behavioral tasks, which would be greater than the similarity between emotion and color concepts. Color represents a sensory domain closely associated with emotion that forms another rich source of descriptive metaphors (Sutton and Altarriba, 2016; Jonauskaite et al., 2020), which, unlike taste, is not as clearly linked to emotion-related neural systems. A recent study of emotion semantics across languages identified that the semantic similarity between emotion concepts is most strongly driven by their similarity in hedonic valence (Jackson et al., 2019). Accordingly, we would also expect that the semantic similarity between taste and emotion concepts would be significantly related to similarity in valence, and that this relationship would be greater than that observed between color and emotion concepts.

## Materials and Methods

### Taste, Emotion, and Color Concepts

A list of 51 English words drawn from a database of cross-linguistic concepts (List et al., 2018) was used to generate the stimuli for the behavioral task, containing 24 words describing emotion concepts and 13 words describing color concepts which were previously used in study of cross-cultural emotion semantics (Jackson et al., 2019). Also included were 14 descriptive concepts related to taste and food properties. The emotion and color concept groups are the same sets of concepts used in a previous study of emotion semantics (Jackson et al., 2019). The Taste concepts consist of 14 concepts selected by the authors (JA, AL, and AM) relating to taste and food qualities, available within the Concepticon database (List et al., 2016). These consist of four of the five basic tastes (sweet, sour, salty, bitter) as well as several food-related concepts identified within the Concepticon database under the Semantic Field of Food and Drink, with the Ontological Category of Property (List et al., 2016^1^). A full list of the concepts for each of these three groups is available in Table 2.

Ratings of valence and arousal for each of these concepts were obtained from the Warriner Affective database, which contains ratings of valence and arousal for over 14,000 English words, obtained from an online survey of an English-speaking population (Warriner et al., 2013). Concreteness (specificity) ratings for these concepts were obtained from the database of concreteness ratings (Brysbaert et al., 2014). Two of the Taste concepts (“cooked”, “unripe”) were not listed in these databases, so those words have been excluded from their respective analyses. We compared each set of concepts on their average levels of valence, arousal, and concreteness using ANOVA.

### Behavioral Experiments

**FIGURE 1:**
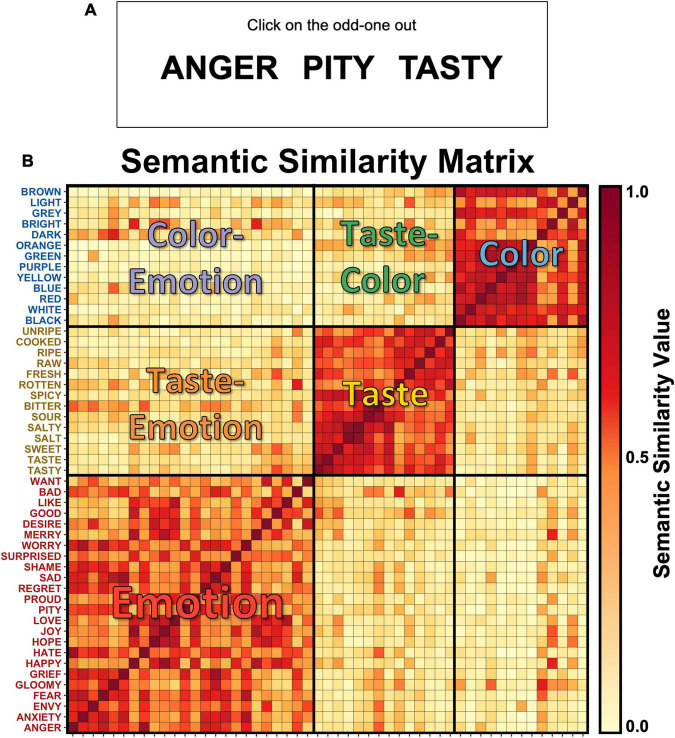
The semantic similarity of Taste, Color, and Emotion concepts. **(A)** In an online behavioral task, we asked participants to select the “odd-one-out” from a series of three-word prompts, each containing concept words related to taste, emotion, or color. **(B)** The data from this task were used to create a similarity matrix, which indicated the semantic similarity of each pair of concepts. The pairwise semantic similarity values in the matrix reflect the observed probability that neither word in a pair was selected as the odd-one-out during the behavioral task.

We examined the semantic structure of taste, color, and emotion concepts behaviorally using an odd-one-out triplet task (Hebart et al., 2020) in a group of online participants on the Amazon Mechanical Turk platform. Ethics approval for this study was granted by the NIH Combined Neuroscience Institutional Review Board under 962 protocol number 93-M-0170. The Institutional Review Board of the NIH approved all procedures, and informed consent was obtained for all online subjects. We selected only English-speaking participants from within the continental United States, who indicated that English was their primary language. Within this task, each subject viewed three words on the screen, randomly drawn from the full set of Emotion, Taste, and Color concepts. For each of these word triplets, subjects were instructed to select the word which was the least similar to the other two concepts (Figure 1A). The choice of the odd-one-out indicates which pair of words participants find to be most similar. For instance, the participants might see the words “GREEN,” “BLUE,” and “SOUR.” As “GREEN” and “BLUE” fall within the same conceptual category, they would most likely pick “SOUR” as the odd-one-out. However, participants might also see the words “GREEN,” “ENVY,” and “UNRIPE,” which all belong to different conceptual categories, so the choice is not so straightforward. One advantage of this task is that, rather than explicitly measuring the similarity of two concepts on one dimension, this task measures the overall implicit similarity of these concepts across multiple dimensions. We can then examine how much one or more specific dimensions (e.g., valence and arousal) contribute to that similarity. Rather than explicitly asking how similar two concepts are, without providing any context for that comparison, in this task the third word in the set always serves as the context by which to compare the other two words. By repeatedly varying that context through this task, we are thus sampling across wide variety of different contexts, which allows us to approximate the implicit similarity of these concepts.

Every possible 3-word combination of these 51 words was tested, which was a total of 20,825 triplets. Each full set of triplets was broken into Human Interaction Tasks (HITs), with 25 distinct triplets per HIT. To verify reliability of the behavioral data, we set out to test the full set of 20,825 triplets twice, then compare the resulting behavioral similarity matrices from those two datasets. We did so by calculating the Pearson’s correlation of the upper triangles of both similarity matrices (see section “Behavioral Data Analysis” for more). The order of those triplets was randomized so that subjects across sessions did not encounter triplets in the same order, thus we tested a total of 1666 unique HITs. Subjects were allowed to complete 2 HITs maximum during a testing session, thus some subjects completed 25 unique triplets and others completed 50 unique triplets. The average duration of a participant’s online session across both datasets was 210 s or 3 min 30 s (SD = 128 s). Subjects were compensated $0.50 for each HIT completed. The triplet set each participant observed was drawn from the same full set of triplets without replacement, participants within each session each completed a unique set of triplets and no participant saw the same triplet twice. The sample size for this study was thus the total number of individuals required to complete all HITs (min 833, max 1666). To filter out any potential non-human responses or non-compliant subjects, we included two catch trial triplets within each HIT which consisted of the words PLUS, MINUS, and EQUALS. For these catch trials, subjects were simply instructed to select the word PLUS. After excluding non-compliant HITs, we re-posted the rejected HITs, so that we could collect responses for all triplet combinations. A total of 1814 HITs were completed across both datasets, by 1166 subjects. Using our exclusion criteria, a total of 148 HITs were rejected for non-compliance (approximately 9%). After excluding non-compliant HITs, the data from a total of 1059 online participants was used for the subsequent analyses. Data were pooled across participants, ignoring any participant-specific effects. Given the number of triplets tested, the data from any individual participant constituted at most 0.12% of the total sample.

### Behavioral Data Analysis

The responses to the triplet task were used to generate a semantic similarity matrix, as follows (Hebart et al., 2020). For every pair of two words from our full set of 51 concepts, we first identified the number of triplets in which both words were present. From this set of triplets, we identified the number of times either word was selected as the odd-one-out. We subtracted this number from the total number of triplets in the set to obtain the number triplets in which neither word was selected. We then divided this number by the number of triplets in the set to obtain the observed probability that neither concept would be selected as the odd-one-out. Higher numerical values of this index thus reflect a greater degree of semantic similarity between the two concepts.

### Concept Groups

We subdivided the upper triangle of the resulting semantic similarity matrix into six sections which included groups of cells containing similarity values comparing concepts within each concept set (Emotion, Taste, and Color groups) or between each concept set (Taste–Emotion, Color–Emotion, and Taste–Color groups; see Figure 1). We then compared the average semantic similarity values within these concept groups in the subsequent analyses.

In our first analysis, we examined whether Taste concepts were more semantically similar to Emotion concepts than Color concepts were to Emotion concepts. For this analysis, we performed an ANOVA comparing the average similarity values of all concept groups. We tested for a main effect of concept group and a main effect of category type (similarity Within concept sets or Between concept sets).

We also performed *post hoc t*-tests comparing the average similarity values for each concept group against every other, including the average value of the cells in the Taste–Emotion group to the average value of the cells in the Color–Emotion group. These *post hoc* statistical tests were then false discovery rate-corrected for multiple comparisons, in order to reduce the likelihood of Type-I errors due to performing multiple statistical comparisons (Benjamini and Hochberg, 1995).

### Relationship to Valence and Arousal

The core dimensions of emotional concepts are valence and arousal (Russell, 2003; Barrett and Bliss-Moreau, 2009), which have been shown to largely account for the variance within semantic networks of emotional concepts (Jackson et al., 2019). One explanation for the relatively greater similarity of taste and emotion concepts, compared to color and emotion concepts, could be that taste and emotion concepts share a similar conceptual structure of valence and arousal. Thus, the similarity between taste and emotion concepts would be much more related to these shared attributes of valence and arousal than would the similarity between color and emotion concepts, the latter of which lack the shared conceptual similarity related to valence and arousal.

To examine this possibility, we used the ratings from Warriner et al. (2013) database to identify the associated valence and arousal of our taste, color, and emotion concepts. We used these ratings to generate valence and arousal similarity matrices for these concepts, with the same dimensions as the semantic similarity matrices, as follows (see Figure 3). For both valence and arousal ratings, we generated separate distance matrices reflecting the Euclidean distance between each pair of concepts. We then rescaled the distances to values to within a range of 0 and 1 by subtracting the minimum distance from each value and then dividing the resulting number by the range of distance values. To generate the final valence and arousal similarity matrices, we subtracted each of these scaled distance values from 1. We subdivided these valence and arousal matrices into six concept groups as we did above with the semantic similarity matrix.

We included these valence and arousal similarity matrices as regressors in general linear models (GLMs) to examine how similarity in valence and arousal is related to semantic similarity of these concepts. We performed an ANCOVA to identify overall effects of valence and arousal as well as significant group × valence and group × arousal interactions, which would signify that the association between similarity in hedonic valence or arousal and semantic similarity differs across concept groups. We then performed *post hoc* tests to examine the valence and arousal relationships for each concept group individually using a GLMs. We specifically compared Taste–Emotion and Color–Emotion groups using ANCOVA to determine if the relationship between valence similarity and semantic similarity differed significantly between these two groups.

We tested the significance of each of these GLMs using a permutation test procedure, in which we randomly permuted the semantic similarity values used as the dependent variable 1000 times before application of the GLM. This allowed us to generate an empirical distribution of random *r*^2^ values, which we then compared to the observed GLM *r*^2^ value. The resulting *p*-value was thus the proportion of values in this empirical distribution greater than our observed value.

## Results

### Behavioral Task Results

The three concept groups differed significantly in average concreteness (*p* < 0.001) and arousal (*p* < 0.001), but not valence (*p* = 0.31). The average triplet response time across both sessions was 5.0 s (SD = 7.5 s). Thus, the average length of a HIT (25 triplets) was approximately 125 s. The average participant session (including 1 or 2 HITs) was 210 s (SD = 128 s). Data from the two complete sets of triplets, which were collected on separate days, were used to construct a pairwise similarity matrix containing values for all 51 × 51 concept pairs. The correlation between the upper triangles of these two pairwise similarity matrices was extremely high (*r* = 0.96, *p* < 0.001), indicating these measurements were extremely stable across multiple groups of participants. The two similarity matrices were averaged together for the subsequent analyses.

**FIGURE 2:**
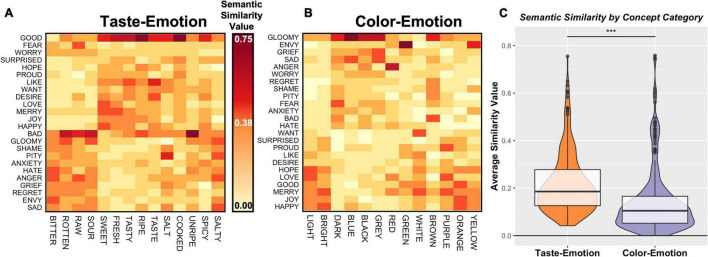
The semantic similarity of Taste and Emotion concept pairs is greater than that of Color and Emotion Concept pairs. We compared the similarity of **(A)** Taste and Emotion concept pairs to **(B)** Color and Emotion concept pairs by selecting the relevant cells of the matrix and comparing the average similarity values within. **(C)** The results of this analysis show that participants judged taste concepts to have greater conceptual similarity to emotion concepts than did color concepts. ***p < 0.001.

As shown in the color-coded heatmap in Figure 1, the individual concepts are tightly clustered within their respective semantic categories. To examine this structure more closely, we next selected the specific arrays within the larger matrix which corresponded to the three within-category partitions (Emotion, Taste, Color) and the three between-category partitions (Taste–Emotion, Color–Emotion, and Taste–Color; see Figure 1B). We then compared the average similarity of the cells in those groups using ANOVA. We observed a main effect of category type, as similarity within category was significantly greater than between-category similarity (*p* < 0.001). Notably, planned *post hoc t*-tests identified that, when comparing between category partitions, the average similarity of taste and emotion concepts was significantly greater than the average similarity of color and emotion concepts (*t* = 7.0, *p* < 0.001; Figure 2; see Table 1 for full set of pairwise comparisons), indicating that pairings of taste and emotion concepts were judged to have greater semantic similarity than pairings of color and emotion concepts. This finding held both for the full set of taste concepts, as well as pairings of the four basic tastes and emotion concepts (*t* = 7.4, *p* < 0.001).

**FIGURE 3:**
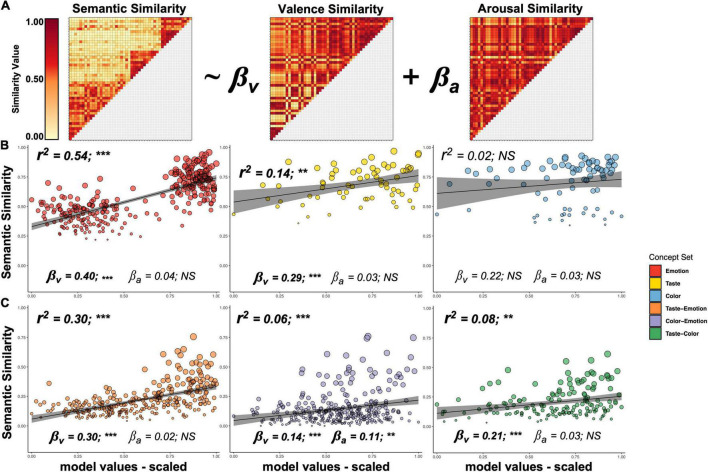
Relationship between semantic similarity and similarity in valence and arousal. **(A)** We compared the pairwise semantic similarity between concepts to those concepts’ similarity in valence and arousal using a general linear model. **(B)** The similarity of Emotion and Taste concepts by themselves, but not Color concepts, was related to their similarity in hedonic valence. **(C)** This relationship with valence was also present in the similarity of Taste–Emotion, Color–Emotion, and Taste–Color concept pairs. β_*v*_, beta-coefficient for valence similarity; β_*v*_, beta-coefficient for arousal similarity.^***^p < 0.001; ^**^p < 0.01; *^NS^p* > 0.05.

**TABLE 1:** Comparison of semantic similarity across different concept groups.

A	B	A mean	B mean	Diff. AB	df	SE	*t*	*P*-value^1^
Emotion	Taste	0.57	0.69	−0.12	117.83	0.02	−5.97	<0.001
Emotion	Color	0.57	0.70	−0.13	120.82	0.02	−5.57	<0.001
Emotion	Taste–Emotion	0.57	0.22	0.35	498.01	0.01	26.47	<0.001
Emotion	Color–Emotion	0.57	0.14	0.42	516.31	0.01	31.90	<0.001
Emotion	Taste–Color	0.57	0.20	0.36	415.60	0.01	25.08	<0.001
Taste	Color	0.69	0.70	−0.01	141.13	0.03	−0.26	0.797
Taste	Taste–Emotion	0.69	0.22	0.47	90.19	0.02	24.47	<0.001
Taste	Color–Emotion	0.69	0.14	0.55	92.20	0.02	28.28	<0.001
Taste	Taste–Color	0.69	0.20	0.48	105.95	0.02	24.10	<0.001
Color	Taste–Emotion	0.70	0.22	0.48	98.10	0.02	21.55	<0.001
Color	Color–Emotion	0.70	0.14	0.55	99.75	0.02	24.89	<0.001
Color	Taste–Color	0.70	0.20	0.49	111.37	0.02	21.46	<0.001
Taste–Emotion	Color–Emotion	0.22	0.14	0.08	597.99	0.01	7.02	<0.001
Taste–Emotion	Taste–Color	0.22	0.20	0.02	333.73	0.01	1.26	0.222
Color–Emotion	Taste–Color	0.14	0.20	−0.06	348.81	0.01	−4.88	<0.001

*^1^p-Values after false discovery rate (FDR) correction for multiple comparisons (Benjamini and Hochberg, 1995).*

**TABLE 2:** Emotion, Taste^a^, and Color concepts.

Concept	Group	Category*	Semantic field*
ANGER	Emotion	Person/thing	Emotions and values
ANXIETY	Emotion	Person/thing	Emotions and values
ENVY	Emotion	Person/thing	Emotions and values
FEAR (FRIGHT)	Emotion	Person/thing	Emotions and values
GLOOMY	Emotion	Property	Emotions and values
GRIEF	Emotion	Person/thing	Emotions and values
HAPPY	Emotion	Property	Emotions and values
HATE	Emotion	Action/process	Emotions and values
HOPE	Emotion	Action/process	Emotions and values
JOY	Emotion	Person/thing	Emotions and values
LOVE	Emotion	Action/process	Emotions and values
PITY	Emotion	Person/thing	Emotions and values
PROUD	Emotion	Property	Emotions and values
REGRET	Emotion	Action/process	Emotions and values
SAD	Emotion	Property	Emotions and values
SHAME	Emotion	Person/thing	Emotions and values
SURPRISED	Emotion	Property	Emotions and values
WORRY	Emotion	Action/process	Emotions and values
MERRY	Emotion	Property	Emotions and values
DESIRE	Emotion	Action/process	Emotions and values
GOOD	Emotion	Property	Emotions and values
LIKE	Emotion	Action/process	Emotions and values
BAD	Emotion	Property	Emotions and values
WANT	Emotion	Action/process	Emotions and values
TASTY	Taste	Property	Food and drink
TASTE (SOMETHING)	Taste	Action/process	Sense perception
SWEET	Taste	Property	Sense perception
SALT	Taste	Person/thing	Food and drink
SALTY	Taste	Property	Sense perception
SOUR	Taste	Property	Sense perception
BITTER	Taste	Property	Sense perception
SPICY	Taste	Property	Food and drink
ROTTEN	Taste	Property	Food and drink
FRESH	Taste	Property	The physical world
RAW	Taste	Property	Food and drink
RIPE	Taste	Property	Food and drink
COOKED	Taste	Property	Food and drink
UNRIPE	Taste	Property	Food and drink
BLACK	Color	Property	Sense perception
WHITE	Color	Property	Sense perception
RED	Color	Property	Sense perception
BLUE	Color	Property	Sense perception
YELLOW	Color	Property	Sense perception
PURPLE	Color	Property	Sense perception
GREEN	Color	Property	Sense perception
ORANGE (COLOR)	Color	Property	Sense perception
DARK	Color	Property	Sense perception
BRIGHT	Color	Property	Sense perception
GREY	Color	Property	Sense perception
LIGHT (COLOR)	Color	Property	Sense perception
BROWN	Color	Property	Sense perception

*Category and semantic field as listed in the Concepticon linguistic database (concepticon.clid.org).

*^a^The “Taste” category includes words referring not only to primary taste qualities but also other non-taste properties of food, such as spiciness, an oral trigeminal sensation.*

### Relationship to Valence and Arousal

Using the Warriner et al. (2013) database, we generated separate pairwise matrices of the similarity of Emotion, Taste, or Color concepts in valence and arousal (Figure 3A). We used the relevant sub-sections of the valence and arousal matrices as regressors in GLMs, to predict the similarity values in our behavioral similarity matrix. An ANCOVA of data from all groups revealed a significant effect of group (*p* < 0.001), valence (*p* < 0.001), arousal (*p* < 0.007), and a significant interaction of group × valence (*p* < 0.001), indicating that the relationship between conceptual similarity and similarity of valence differed across concept groups. We did not observe a significant group × arousal interaction effect (*p* = 0.57). At the individual concept group level, we observed that the semantic similarity of Emotion concepts was significantly related to their similarity in valence (β_*v*_ = 0.40, *p* < 0.001; see Figure 3B) as it was for Taste concepts (β_*v*_ = 0.29, *p* < 0.005) but not Color concepts (β_*v*_ = 0.22, *p* = 0.25). For the between-category concepts, we observed that the similarity of Taste–Emotion concepts was also highly related to their similarity in valence (β_*v*_ = 0.30, *p* < 0.001; Figure 3C), as was the similarity of Taste–Color concepts (β_*v*_ = 0.21, *p* < 0.001) and, to a somewhat lesser degree, to Color–Emotion concepts (β_*v*_ = 0.14, *p* < 0.001). Importantly, when comparing the valence relationship between Taste–Emotion and Color–Emotion groups, we observed a significant group × valence interaction (*p* < 0.001), as the relationship between semantic similarity and valence similarity was significantly greater for Taste–Emotion than Color–Emotion (*t* = 3.3, *p* < 0.001). Again, this finding held both for the full set of Taste–Emotion concept pairs, as well as for pairings of the four primary taste qualities and emotion concepts (*t* = 3.4, *p* < 0.001). Similarity in arousal scores was not significantly related to conceptual similarity in any of the concept groups except for the Color–Emotion group (β_*a*_ = 0.11, *p* = 0.004).

## Discussion

We sought to understand the prevalence of verbal taste metaphors for emotional concepts by using a behavioral task designed to test the semantic similarity of a variety of concepts related to taste, emotion, and color. We found that taste concepts are judged to be more semantically similar to emotion concepts than are color concepts, another rich and ubiquitous source of metaphors for emotional concepts (Sutton and Altarriba, 2016; Jonauskaite et al., 2020).

We next examined the associated valence and arousal of these concepts and confirmed that the similarity of emotion concepts was highly related to their similarity in valence, a relationship which accounted for over half of the variability in their semantic similarity. This finding underscores the importance of hedonic valence as one of the core features of emotional experience (Russell, 2003; Barrett and Bliss-Moreau, 2009) and echoes the prior finding that hedonic valence and arousal represent a source of universal structure for emotion semantics (Jackson et al., 2019). We also found that the similarity of taste and emotion concepts was also significantly related to their similarity in valence, which accounted for nearly 30% of the variance in the distribution (see also Zhou and Tse, 2020). This relationship between valence and semantic similarity was also significantly greater between taste and emotion concepts than between color and emotion concepts. These findings support our prior prediction that a shared ability to communicate valence underlies the similarity in conceptual structure between these two distinct conceptual domains.

Conceptual metaphor and grounded cognition theories imply that these conceptual domains are tied together not just in the structure of the English language, but in the structure of cognition as well (Lakoff and Johnson, 1980; Barsalou, 2008; Winter, 2016; Speed and Majid, 2020). Thus, the association between taste and emotion concepts may be due, in part, to the recruitment of similar neural structures involved in processing these separate conceptual domains, such as the insular cortex (Singer et al., 2009; Small, 2010; Avery et al., 2017, 2020). In support of this possibility, an fMRI study of metaphorical statements identified that when metaphorical taste expressions were substituted for emotion expressions, they led to increased activation of the insular and orbitofrontal cortices (Citron and Goldberg, 2014), two regions commonly associated with the perception of taste and inferred taste when viewing pictures of appetizing foods (Simmons et al., 2013b,^2014^; Avery et al., 2020, 2021), as well as the amygdala and hippocampus. This suggests that the substitution of more concrete metaphorical expressions makes these statements more emotionally engaging and thus better mechanisms for conveying their intended emotional information.

Interestingly, though the similarity of color concepts was not related to valence or arousal, the similarity between color concepts and emotion concepts was related to these factors, though to a degree much less than taste and emotion concepts. Previous studies have also identified valence and arousal related associations between color and emotion words (Sutton and Altarriba, 2016). Other studies suggest that this pattern is not limited to linguistic metaphors, but that similarity in hedonic valence may underly cross-modal correspondence between color and music (Palmer et al., 2013), color and taste (Wan et al., 2014), as well as taste and abstract geometric shapes (Velasco et al., 2016; Wang and Spence, 2017). Thus, it is not surprising that, in the present study, we also identified that the similarity of taste and color concepts was also related to their similarity in valence. Taken together, these results suggest that a similarity in hedonic valence may be a key component of any metaphorical association with emotion concepts, as well as other concept groups that contain a high affective internal structure.

One limitation of the present study is that our behavioral data as well as the valence and arousal norms we employed in the analysis of this data (Warriner et al., 2013) were both obtained from western, English-speaking populations. Taste–emotion metaphors are present across multiple languages, however (for example, see Zhou and Tse, 2020), implying the association between these two domains might be a universal feature of human language. Thus, it will be important for future studies to obtain more widespread cross-cultural measures of these norms and associations (see Jonauskaite et al., 2020) in order to verify their similarity across human languages (see Kemmerer, 2019 for an in-depth discussion of culture and conceptual structure). Future studies on the association between sensory and emotional concepts using this task design would also benefit from a broader group of sensory concept categories, such as olfaction, which also has a close association with emotion at the neural level (Zald and Pardo, 1997; Rolls, 2008; Kontaris et al., 2020).

Some of the concepts used in the “taste” category in this study have multiple sensory associations beyond strictly taste, such as olfactory, tactile, or oral trigeminal qualities, especially the food-related properties such as “spicy” and “rotten.” Within chemosensory research, the concept of “taste” refers strictly to the five basic tastes (sweet, salty, sour, bitter, and umami/savory). However, within this study, we sought to investigate the more common concept of taste as present within the English language, which is often interchangeable with flavor (the combination of taste and smell). Notably, human neuroimaging studies have identified that food-related properties such as flavor, texture, and spiciness recruit the same gustatory regions of the insula (de Araujo and Rolls, 2004; Seubert et al., 2015; Kawakami et al., 2016) that are involved in the response to basic tastes (Avery et al., 2020). As this region also responds strongly to visual representations of foods in a taste–quality-specific manner (Avery et al., 2021), the conceptual processing of these various food-related properties likely involves the same neural substrate in the brain. Importantly, we identified that the core results of this study, that of greater semantic similarity for Taste–Emotion pairings and the greater relationship between semantic similarity and valence similarity for Taste–Emotion pairings, held for the four primary taste qualities as well as for the full set of Taste concepts.

Conceptual metaphors, in general, are used as a method for communicating one concept through another related concept (Lakoff and Johnson, 1980). When the concepts to be conveyed are more abstract and internal, such as emotion concepts, conceptual metaphors from more concrete domains, such as taste or color, may be used to ground the concepts in experiences from our external shared environment (Lakoff and Johnson, 1980, 1999; Barsalou, 2008; for a review of conceptual grounding in touch, taste, and smell see Speed and Majid, 2020). This process of conceptual grounding may then result in a conceptual structure for these abstract concepts which is more easily shared between individuals. Our findings suggest that within this process of conceptual metaphor generation, we use the core affective properties of valence and arousal as a way of bridging the divide between concepts relating to our shared sensory environment and concepts which relate to our internal experience.

## Data Availability Statement

The datasets presented in this study can be found in online repositories. The names of the repository/repositories and accession number(s) can be found below: https://osf.io/njch2/.

## Ethics Statement

The studies involving human participants were reviewed and approved by the NIH Combined Neuroscience Institutional Review Board. The patients/participants provided their written informed consent to participate in this study.

## Author Contributions

JA and AM designed the research. JA, AL, and MC performed the research. JA analyzed the data. JA, AL, MC, and AM wrote the manuscript. All authors contributed to the article and approved the submitted version.

## Conflict of Interest

The authors declare that the research was conducted in the absence of any commercial or financial relationships that could be construed as a potential conflict of interest.

## Publisher’s Note

All claims expressed in this article are solely those of the authors and do not necessarily represent those of their affiliated organizations, or those of the publisher, the editors and the reviewers. Any product that may be evaluated in this article, or claim that may be made by its manufacturer, is not guaranteed or endorsed by the publisher.

## Footnotes

1
http://concepticon.clld.org/
